# Pathological Steps of Cancer-Related Lymphedema: Histological Changes in the Collecting Lymphatic Vessels after Lymphadenectomy

**DOI:** 10.1371/journal.pone.0041126

**Published:** 2012-07-24

**Authors:** Makoto Mihara, Hisako Hara, Yohei Hayashi, Mitsunaga Narushima, Takumi Yamamoto, Takeshi Todokoro, Takuya Iida, Naoya Sawamoto, Jun Araki, Kazuki Kikuchi, Noriyuki Murai, Taro Okitsu, Iori Kisu, Isao Koshima

**Affiliations:** 1 Department of Plastic and Reconstructive Surgery, The University of Tokyo, Tokyo, Japan; 2 Department of Vascular Surgery, Saiseikai Kawaguchi General Hospital, Saitama, Japan; 3 Department of Rehabilitation Medicine, Keio University, Tokyo, Japan; 4 Department of Obstetrics and Gynecology, Keio University School of Medicine, Tokyo, Japan; Children’s Hospital Boston, United States of America

## Abstract

**Introduction:**

To date, an electron microscopy study of the collecting lymphatic vessels has not been conducted to examine the early stages of lymphedema. However, such histological studies could be useful for elucidating the mechanism of lymphedema onset. The aim of this study was to clarify the changes occurring in collecting lymphatic vessels after lymphadenectomy.

**Methods:**

The study was conducted on 114 specimens from 37 patients who developed lymphedema of the lower limbs after receiving surgical treatment for gynecologic cancers and who consulted the University of Tokyo Hospital and affiliated hospitals from April 2009 to March 2011. Lymphatic vessels that were not needed for lymphatico venous anastomosis surgery were trimmed and subsequently examined using electron microscopy and light microscopy.

**Results:**

Based on macroscopic findings, the histochemical changes in the collecting lymphatic vessels were defined as follows: normal, ectasis, contraction, and sclerosis type (NECST). In the ectasis type, an increase in endolymphatic pressure was accompanied by a flattening of the lymphatic vessel endothelial cells. In the contraction type, smooth muscle cells were transformed into synthetic cells and promoted the growth of collagen fibers. In the sclerosis type, fibrous elements accounted for the majority of the components, the lymphatic vessels lost their transport and concentrating abilities, and the lumen was either narrowed or completely obstructed.

**Conclusions:**

The increase in pressure inside the collecting lymphatic vessels after lymphadenectomy was accompanied by histological changes that began before the onset of lymphedema.

## Introduction

Limb lymphedema, which develops after lymphadenectomy for cancer treatment, is an increase in the volume of the diseased limb caused by the subcutaneous accumulation of a protein-rich fluid [1], [2]. Because the lymphatic fluids can no longer be transported, the pressure in the collecting lymphatic vessels in the limbs gradually increases, the flow of lymph slows down [3]–[5], and lymphedema eventually develops. When the duration of illness is prolonged, the lymphedema may develop into lymphangiosarcoma. The life expectancy of a patient with this condition is limited to a few months to 2 years [6], [7].

Currently, the mechanism underlying the onset of lymphedema is unknown, and a treatment has yet to be established for preventing the onset of this disease. However, once lymphedema has become established, complex physical therapy is said to be the gold standard for its treatment [8]–[11]. In addition, surgical treatments such as lymphatic venous anastomosis (LVA) [12], [13] and lymph node transplantation [14] have recently been developed, expanding the treatment options for those cases in which complex physical therapy alone is insufficient. However, the indications and non-indications of surgical treatment have not been well defined [15].

The collective lymphatics, which play a central role in the onset of lymphedema, show a relatively simple organization. Like veins, the collecting lymphatics display a trilaminar structure, consisting of an internal, medial, and outer membrane. The internal layer consists of endothelial cells; the medial layer, varying proportions of smooth muscle; and the external layer, an extracellular matrix containing fine bundles of collagen and elastin fibers. A fat-rich adventitia incorporating microcirculation is also present [16].

An electron microscopy study to examine collecting lymphatic vessels of the early stages lymphedema has not been performed. However, such studies could be useful for the elucidation of the mechanism of the lymphedema onset, the establishment of a therapeutic method, and the development of preventive methods. In this study, we investigated the changes occurring in collecting lymphatic vessels after lymphadenectomy by performing electron and light (immunostaining) microscopic studies of collecting lymphatic vessels in the human body.

The aim of this study was to clarify the changes that occurred in patients’ collecting lymphatic vessels after lymphadenectomy until early after the onset of lymphedema.

**Table 1:** Case summary.

Number of cases (sample number)	37 (114)
Age/yr (average)	32–75 (56.2)
Lymphedema latent period/month (average)	8–81 (36.9)
Lymphedema morbidity period/month (average)	9–69 (34.4)
No. of subjects with respective lymphedema severity (%)012a2b3	4512106
Primary diseasecervical cancerendometrial cancerovarian cancer	2872
RadiotherapyNoYes	2710

**Table 2:** (Online only) Classification of the types of collecting lymphatic vessels and the macroscopic anatomical findings (NECST).

	Normal Type (Step 0)	Ectasis Type (Step 1)	Contraction Type (Step 2)	Sclerosis Type (Step 3)
Vaso lymphaticum	(++)	(+)	(−)	(−)
Lymphangiectasia	(−)	(+)	(−)	(−)
White turbidity and thickening of the lymphatic vessel wall	(−)	(−)	(+)	(++)
Narrowing of the lumen	(−)	(−)	(−)	(+)

## Methods

### Patients

Histological evaluation (immunostaining and electron microscopic examination) of collecting lymphatic vessels was performed on 114 specimens from 37 patients who developed lymphedema of the lower limbs after surgical intervention for gynecologic cancers and who consulted the University of Tokyo Hospital and Saiseikai Kawaguchi General Hospital from April 2009 to March 2011 (Table 1). Upper limb lymphedema patients were excluded because the rate of progression is different between lower and upper limb lymphedemas as a consequence of gravity. The experiments performed in this study were conducted with the approval of the ethical committees at the University of Tokyo and Saiseikai Kawaguchi General Hospital. All the patients provided written informed consent. We examined 28 cases of cervical cancer, 7 cases of endometrial cancer, and 2 cases of ovarian cancer. The staging of lymphedema was performed according to the classification system of the International Society of Lymphology (ISL). Stage 0 refers to a latent or subclinical condition where swelling is not evident despite impaired lymph transport. Stage I represents an early accumulation of fluid that is relatively high in protein content and that subsides with limb elevation. Moreover, pitting may occur at this stage. Stage II signifies that limb elevation alone rarely reduces tissue swelling; pitting is manifest. Late in Stage II, the limb may or may not pit as tissue fibrosis supervenes. Stage III encompasses lymphostatic elephantiasis in which pitting is absent and trophic skin changes such as acanthosis, fat deposits, and warty overgrowths develop. Stage 0 accounted for 4 cases (number of samples: 15); Stage I, 5 cases (18); Stage IIa, 12 cases (36); Stage IIb, 10 cases (34); and Stage III, 6 cases (11).

**Figure 1:**
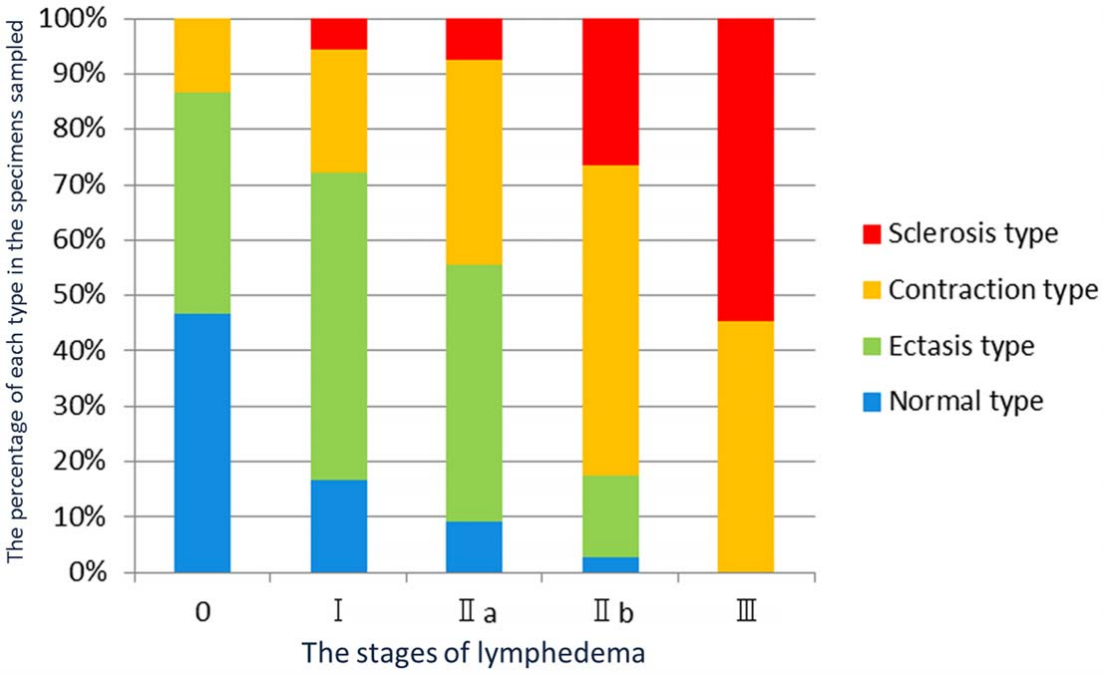
Association between the classification of the types of collecting lymphatic vessels and the staging of lymphedema. The horizontal axis shows the stages of lymphedema. The vertical axis shows the percentage of each type in the specimens sampled at each stage of the disease.

### Collection of Samples

The tests were conducted using collecting lymphatic vessels that did not need to be anastomosed and were trimmed during lymphatic venous anastomosis surgery. Samples were collected from the diseased limb, namely from the medial sides of the thigh, knee, or ankle. In addition, in the normal control group, collecting lymphatic vessels were sampled from intestinal membranes trimmed during free jejunal transplantation.

**Figure 2:**
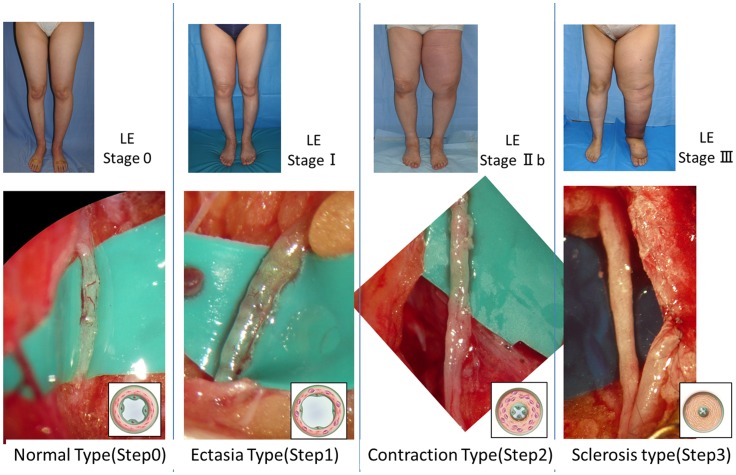
Staging of lymphedema and the macroscopic anatomical findings in the collecting lymphatic vessels associated with the stages. In the normal type, microvascular networks were found to nourish the largely developed walls of the collecting lymphatic vessels. The microvascular networks were gradually lost with the progression of the disease stages. In addition, the lymphatic vessel lumen was found to be dilated in the ectasis type, which was associated with an increase in endolymphatic pressure. Because increases in smooth muscle cells and collagen fibers are the major causes of the cloudiness and thickening of the lymphatic vessel wall, they were found to be prominent in the contraction type and the sclerosis type. LE: lymphedema.

### Classification of the Types of Collecting Lymphatic Vessels Based on Intraoperative Macroscopic Findings

In this study, we classified the macroscopic findings observed under an operating microscope into 4 types: the normal, ectasis, contraction, and sclerosis types (NECST). The details of the classification based on the macroscopic findings are shown in Table 2. We classified the types of collecting lymphatic vessels found at each stage of lymphedema and then examined the correlations among the types and stages by dividing each type by the total number of samples collected at each stage of disease.

**Figure 3:**
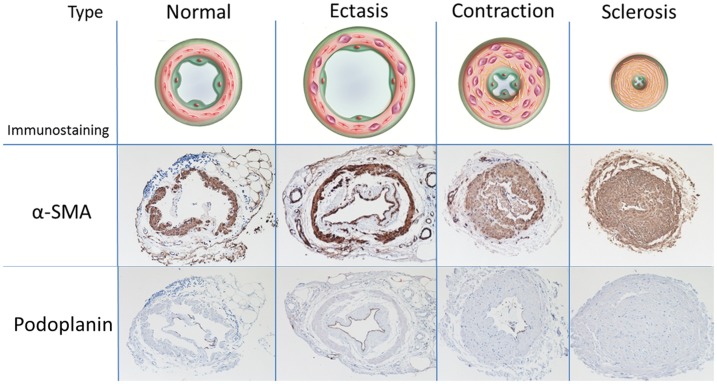
Classification of the types of collecting lymphatic vessels and the corresponding immunostaining findings. The first row: Findings from alpha- smooth muscle actin (αSMA) staining. In the normal type, several layers of smooth muscle cells were present. In the ectasis type, the smooth muscle cell layer was moderately thin. In the contraction type, a very thick layer of smooth muscle was present directly beneath the endothelium, and the lumen was narrowed to a certain degree. In the sclerosis type, the lumens were narrowed. The second row: Findings from podoplanin staining. The normal type was positive for podoplanin staining in the endothelium; the ectasis type was strongly positive; the contraction type, slightly positive; the sclerosis type, negative.

### Histological Examination

The lymphatic vessels were stained with hematoxylin-eosin and examined under the microscope. In addition, immunostaining was performed using podoplanin mouse monoclonal antibody (Nichirei Biosciences, 413451, Tokyo, Japan) and α-SMA mouse monoclonal antibody (DakoCytomation, M0851) in order to determine the expression of the corresponding vascular histological markers. Podoplanin, a glomerular podocyte membrane mucoprotein, has been shown to localize to the endothelium of lymphatic vessels [17].

### Sample Processing for Scanning Electron Microscopy (SEM)

The lymphatic vessels were fixed in 2.5% glutaraldehyde in phosphate-buffered saline at 4°C for 1 week, and then washed in phosphate buffer to remove the fixative solution. Next, the samples were post-fixed in 1% OsO_4_ and washed in phosphate buffer. Afterwards, the samples were dehydrated in serially increasing concentrations of ethanol (50–100%). The ethanol was then substituted with mixed solutions of 100% ethanol and 3-methylbutyl acetate. Critical point drying of the samples was subsequently performed using a JCPD-5 (JEOL, Tokyo, Japan). The specimens were placed in specimen blocks and subjected to conductive coating (Pt) with a Precision-etching coating system (Model 682; Gatan, CA, USA). The samples were examined with a JSM-7001F electron microscope (JEOL, Tokyo, Japan).

**Figure 4:**
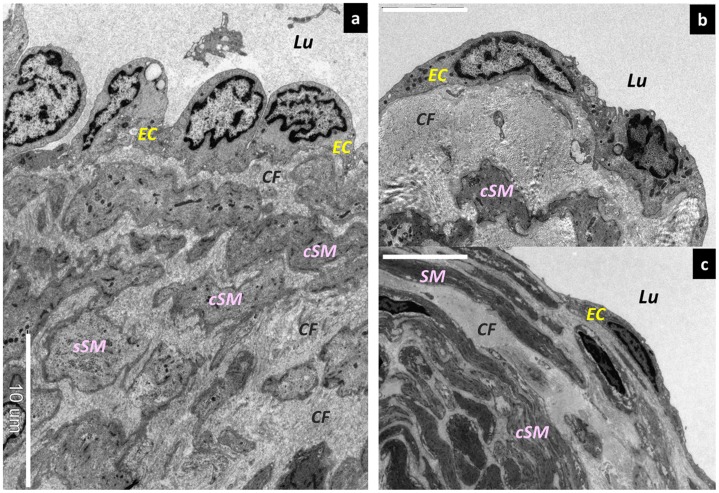
Morphological changes in lymphatic vessel endothelial cells. Scanning electron microscopy (SEM) findings. (a) Lymphatic vessel endothelial cells protruding into the lymphatic lumen in the normal type. Bar = 10 µm. (b) Lymphangiectasia at its early stages. The lymphatic vessel endothelial cells are slightly flattened. Bar = 5 µm. (c) Medium-stage lymphangiectasia. The lymphatic vessel endothelial cells are markedly flattened. Bar = 10 µm. Lu; Lumen, EC; endothelium cells, sSM; synthetic smooth muscle cells, cSM; contractile smooth muscle cells, CF; collagen fibers.

**Figure 5:**
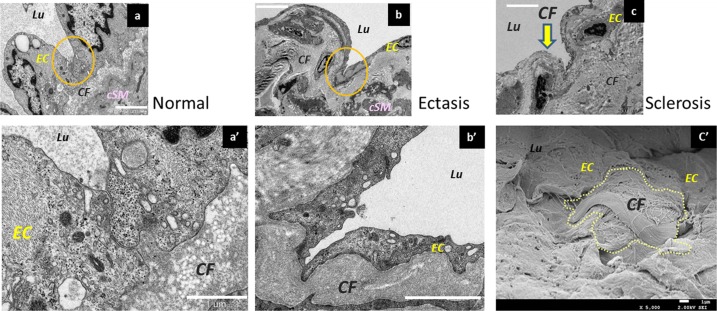
Changes in the desmosomes of lymphatic vessel endothelial cells as shown by electron microscopy. (a) Desmosomes in the normal type. In a three-dimensional structure, they adhere to the adjacent lymphatic vessel endothelial cells. Bar = 2 µm. (a’) Magnified image. Bar = 2 µm. (b) Desmosomes of lymphatic vessel endothelial cells in the ectasis type. The cell-to-cell adhesions have gradually loosened and disassembled, so that the cells adhere only at points. Bar = 10 µm. (b’) Magnified image. Bar = 2 µm. (c) Lymphatic endothelial cells in the collecting lymphatic vessels in the sclerosis type. The desmosomes are disassembled, and the collagen fibers under the lymphatic vessel endothelium are exposed in the lymphatic vessel lumen (yellow arrow). Bar = 5 µm. (c’) Collagen fibers exposed in the lymphatic vessel lumen (Electron microscopy, SEM). Lymphatic vessel endothelial cells are missing, and fascicles of collagen fibers are exposed in the lymphatic vessel lumen. Bar = 1 µm. EC; endothelium cells, sSM; synthetic smooth muscle cells, cSM; contractile smooth muscle cells, CF; collagen fibers.

### Sample Processing for Transmission Electron Microscopy (TEM)

The samples were processed in the same manner as those for SEM from the fixation step through the dehydration steps involving ethanol. After the ethanol was substituted with propylene oxide (PO), the samples were infiltrated with epoxy resin [a mixture of Epon812, dodecenyl succinic anhydride, methyl nadic anhydride, and 2,4,6-tri(dimethylaminomethyl) phenol] using a series of mixed solutions of PO and epoxy resin and then epoxy resin alone. The samples were polymerized for 3 days (35°C to 60°C). The samples were sectioned at 80 nm, and the resultant ultrathin sections were prepared using conventional staining with uranyl acetate and lead citrate. The sections were then examined with a JEM-1011 electron microscope (JEOL, Tokyo, Japan).

**Figure 6:**
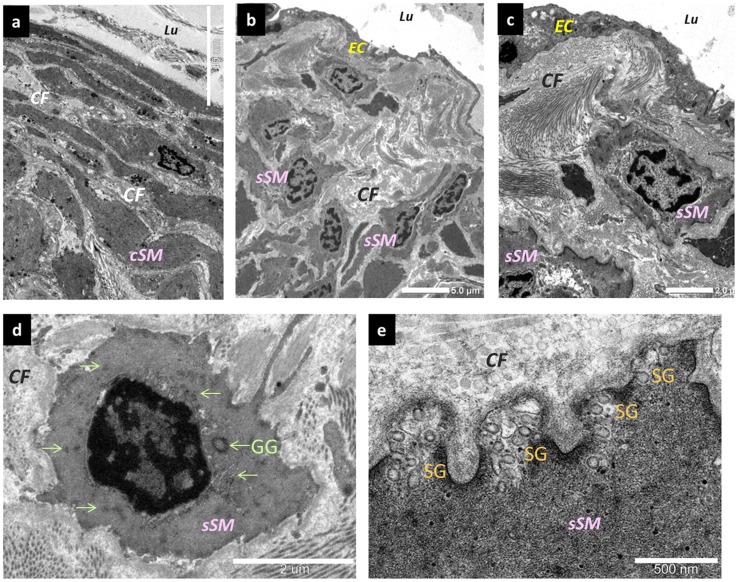
Changes in smooth muscle cells. (a) Normal type. They are present in the medial layer of the collecting lymphatic vessels, as contractile smooth muscle cells. Synthetic smooth muscle cells are only partially present. Bar = 10 µm. (b) Changes affecting the smooth muscle cells in the contraction type. Synthetic smooth muscle cells migrated directly beneath the endothelial cells and proliferated. Bar = 5 µm. (c) Synthetic smooth muscle cells stimulated collagen fiber hyperplasia directly beneath the lymphatic vessel endothelial cells. Bar = 2 µm. (d) Synthetic smooth muscle cells. An increase in glycogen granules inside the cells is evident. (e) The periphery of synthetic smooth muscle cells. Secretory granules are present in large numbers. Bar = 500 nm. EC; endothelium cells, sSM; synthetic smooth muscle cells, cSM; contractile smooth muscle cells, CF; collagen fibers, GG; glycogen granules (green arrows), SG; secretory granules.

## Results

A strong association was found between our proposed classification for the types of collecting lymphatic vessels at each stage of lymphedema (NECST) and the staging classification of lymphedema (Figure 1). With advancing stages of lymphedema, a lower number of normal types were found among the collected samples. No normal type was sampled in stage III, the most severe stage of the disease. In contrast, the sclerosis type gradually increased in number with the progression of the disease and was the one primarily collected in stage III. The ectasis type was commonly found in mild cases, such as in stages I and II of the disease, whereas the contraction type was found to be common in severe cases, such as in stages II and III. Thus, collecting lymphatic vessels gradually transformed from one type to the next.

**Figure 7:**
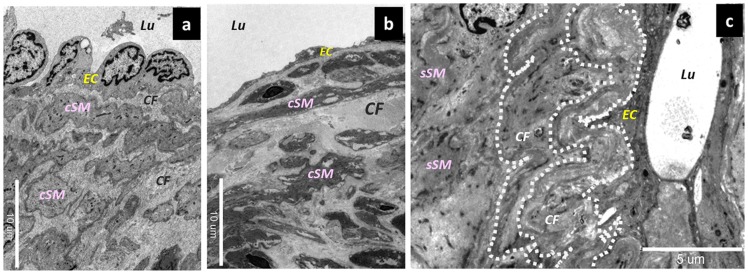
Changes affecting collagen fibers in collecting lymphatic vessels. (a) Collagen fibers in the normal type. Contractile smooth muscle cells and collagen fibers were mixed together in the medial layer. Bar = 10 µm. (b) Collagen fibers in the ectasis type. The dilation of the lymphatic vessel was accompanied by an elongation of collagen fibers, causing the slightly thickened aspect. Bar = 10 µm. (c) Collagen fibers in the contraction type. Once the collagen fibers were elongated, they became multilayered and tortuous (dotted line), in association with the hypertrophy of the walls of the collecting lymphatic vessels. Bar = 5 µm. EC; endothelium cells, seSM; synthetic smooth muscle cells, sySM; synthetic smooth muscle cells, cSM; contractile smooth muscle cells, CF; collagen fibers.

**Figure 8:**
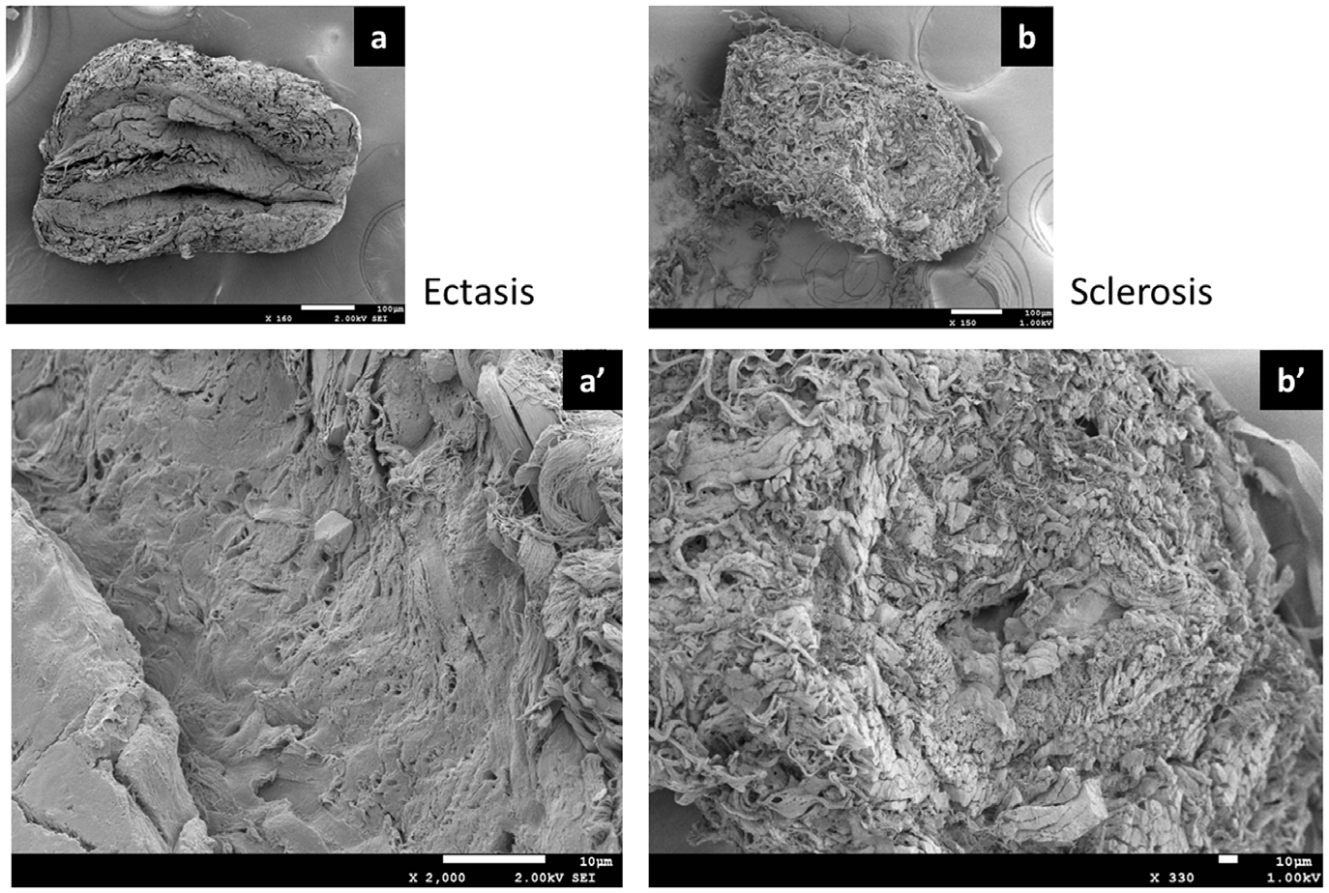
Electron microscopic and SEM findings. (a) Normal type. Bar = 100 µm. (a’) Ectasis type. The lymphatic vessel lumen was dilated and entirely covered with lymphatic vessel endothelial cells. Bar = 10 µm. (b) Sclerosis type. Bar = 100 µm. (b’) The lymphatic vessel wall was thickened because of the hyperplasia of smooth muscles and collagen fibers. The lymphatic vessel lumen was narrowed and as shaped like a pinhole. Bar = 10 µm.

The appearance of the lymphatic vessels associated with each stage of lymphedema is shown in Figure 2. In the normal type, microvascular networks coiling around the lymphatic walls were found to nourish the collecting lymphatic vessels. The microvascular networks were gradually lost with the progression of the disease stages. In addition, the lymphatic vessel lumen was found to be dilated in the ectasis type, which was associated with an increase in endolymphatic pressure. Increases in smooth muscle cells and collagen fibers have been found to be the major causes of the cloudiness and thickening of the lymphatic vessel wall and were observed to be prominent in the contraction type and the sclerosis type. As the lymphedema progressed, the lymphatics became harder, lost elasticity, and seemed to lose the ability to move peristaltically to transport lymphatic fluid.

### Light Microscopic Findings

To examine the lymphatic endothelial cells, we stained for the endothelial protein podoplanin (Figure 3). The normal type was found to be positive for podoplanin staining in a layer of the endothelium of the collecting lymphatic vessels. The ectasis type was found to be strongly positive; the contraction type, slightly positive; the sclerosis type, negative. We also used α-SMA staining to examine the smooth muscle cells. In the normal type, several layers of smooth muscle cells were present in a circular disposition in the medial layers of the collecting lymphatic vessels. In the ectasis type, as an adaptation to the dilatation of the lymphatic vessels, the smooth muscle cell layer in the medial layer was moderately thin in some cases. Like the normal type, several layers of smooth muscle cells were present. In the contraction type, a very thick layer of smooth muscles was present directly beneath the endothelium of the lymphatic vessels, and an increase in the size of the smooth muscle layer was accompanied by a narrowing of the lumen of the lymphatic vessels. In the sclerosis type, the lumens were narrowed in such a way that they were shaped like pinholes. In some cases, they were completely occluded.

### Electron Microscopic Findings

In terms of the endothelial cells in the lymphatic vessels, those in the normal type were found to protrude toward the lymphatic vessel lumen (Figure 4a). In contrast, the endothelial cells in the ectasis type were found to be smooth and did not protrude into the lumen (Figure 4 b, c). This condition is known as “lymphangiectasis.” The focal adhesions (desmosomes) between the endothelial cells adhered strongly and widely in the normal type but were weakened and adhered only partially in the ectasis type (Figure 5 a, b). In the sclerosis type, the adhesion between the lymphatic vessel endothelial cells was completely disassembled, and the collagen fibers beneath the lymphatic vessel endothelial cells were exposed in the lymphatic vessel lumen (Figure 5c).

In terms of the smooth muscle cells, those in the normal type were present in the medial layer of the collecting lymphatic vessels as contractile smooth muscle cells, which are an usual type of differentiated smooth muscle cell (Figure 6a). Synthetic smooth muscle cells, which have increased rates of proliferation, migration, and production of extracellular matrix [18], were also present in small numbers in the medial layer of the normal type. Moreover, the synthetic smooth muscle cells had follicles present in the cytoplasm. In the contraction type, the synthetic smooth muscle cells had migrated directly beneath the lymphatic vessel endothelial cells and had proliferated (Figure 6b). In addition, these muscle cells had also promoted the hyperplasia of the collagen fibers directly beneath the lymphatic vessel endothelial cells (Figure 6c, d, e). Moreover, the synthetic smooth muscle cells had proliferated in the medial layer, were present between the contractile smooth muscle cells, and had promoted the proliferation of collagen fibers. Glycogen granules and secretory granules were increased in abundance inside the synthetic smooth muscle cells.

In terms of the collagen fibers, those in the normal type were present in the medial layer (Figure 7a). Meanwhile, in the contraction type, the synthetic smooth muscle cells had directly migrated into the lumen beneath the lymphatic vessel endothelial cells (Figure 7b). This led to the formation of a thick layer of collagen fibers in the lumen. The thick layer of collagen fibers became tortuous and stratified in association with the changes in the collecting lymphatic vessels, which had shifted from the expansion phase to the contraction phase and finally to the fibrosis stage (Figure 7c). In the sclerosis type, the lymphatic vessel wall was thickened because of the hyperplasia of the smooth muscles and collagen fibers. Further, the lymphatic vessel lumen was narrowed and shaped like a pinhole (Figure 8).

## Discussion

This study has revealed that the progression of disease in limb lymphedema is accompanied by 1) the development of a smoothing of the endothelial cells in the collecting lymphatic vessels, 2) a disassembly of focal adhesions (desmosomes), 3) a growth and transformation of smooth muscle cells, 4) a thickening of the basal membrane, and 5) a proliferation of collagen fibers. This is the first study to report the changes in the endothelial cells and basal membrane of the collecting lymphatic vessels, transformation of smooth muscle cells, and proliferation of collagen fibers during lymphedema.

To date, only a small number of reports have examined the changes affecting normal tissues and collecting lymphatic vessels in limb lymphedema, so that the pathological changes in lymphedema remain unknown. In 1999, in a study using electron microscopy, Koshima *et al.* reported having found smooth muscle cell atrophy in the collecting lymphatic vessels of patients with terminal lymphedema [19]. In a study using immunostaining analysis, Ogata *et al.* reported the possibility of the transformation of smooth-muscle cells in collecting lymphatic vessels [20]. Our report is the first one regarding the progressive changes that occur in collecting lymphatic vessels in mild or moderate lymphedema after lymphadenectomy.

The lumen of a collecting lymphatic channel is normally covered by a layer of endothelial cells. The endothelial cells are completely wrapped by the basal membrane, which is surrounded by smooth muscle cells and fibrous elements. A large number of nutrient vessels and unmyelinated nerves penetrate the lymphatic vessel from the side of the adventitia and up to the smooth muscles directly beneath the endothelium. In addition, a number of tricuspid valves are present and form a one-way flow directed toward the venous angle [21], [22].

Recently, slow spontaneous contractions have been found to occur in lymphatic vessels at a frequency of 2–4 times per minute; and these active contractions have been found to serve as the driving force for lymphatic transport [23]–[25]. The conditions that facilitate the occurrence of active contractions include a very high smooth muscle content in the lymphatic vessel wall, the presence of large amounts of mitochondria and glycogen granules around the nucleus, and the penetration of feeding vessels into the smooth muscle layer directly beneath the endothelium. The functions of the collecting lymphatic vessels are related to their transport and concentrating abilities. In this study, we have classified the changes in collecting lymphatic vessels into 4 different types.

The normal type consists of healthy collecting lymphatic vessels. The collecting lymphatic vessels are divided into 3 layers, and lymphatic endothelial cells surround the entire circumference of the lymphatic vessel lumen. Collagen fibers and smooth muscles are present in the medial layer.

The ectasis type is a condition that is found early after lymphadenectomy. In collecting lymphatic vessels that have lost their downstream opening after lymphadenectomy, the endolymphatic pressure increases markedly, and the lymph flow becomes slow. Histologically, the increase in endolymphatic pressure was accompanied by a dilatation in the lumen of the collecting lymphatic vessels and by a flattening of the lymphatic vessel endothelial cells. The intercellular attachments (desmosomes) between the endothelial cells also gradually changed from strong to weak. Eventually, these intercellular attachments will be lost, and the fibrous elements will become exposed in the lumen of the collecting lymphatic channel. Further, the smooth muscles in the medial layer underwent a transformation, with gradually differentiating smooth muscle cells changing into synthetic smooth muscle cells. As the lymphatic vessel wall dilated, the collagen fibers become thin and elongated.

In the contraction type, the constant increase in the endolymphatic pressure leads to compensatory histological changes that mainly affect the smooth muscle cells. Due to the sustained elevation of the endolymphatic pressure, the lymphatic vessel endothelial cells remain smooth. In terms of the smooth muscle cells, some of the transformed synthetic smooth muscle cells migrated directly beneath the endothelial cells, promoting the proliferation of collagen fibers. This resulted in the formation of thick collagen fibers that become mixed with the smooth muscle cells in the medial layer. Consequently, contractile smooth muscle cells would have difficulty contracting because of hindrance by the thick collagen fibers, resulting in a functional decline in the collecting lymphatic vessels. The same applies for the synthetic smooth muscle cells, since the increasing amounts of smooth muscle cells and collagen fibers were accompanied by an increase in the amount of elastic fibers between the smooth muscle cells.

In the sclerosis type, the intercellular attachments between the lymphatic endothelial cells were lost, causing the lymphatic fluid flowing inside the collecting lymphatic vessels to leak outside. The pressure in the collecting lymphatic vessels is maintained at high levels in some cases but is low in others because of the leakage of the lymphatic fluid. This type is characterized by markedly increased smooth muscle cells and collagen fibers. However, as cellulitis developed, the amount of smooth muscle cells in the collecting lymphatic vessels decreased, and the fibrous elements become the major components. As a consequence, both the transport ability and the concentrating ability become lost. The lymphatic vessel lumen was either markedly narrowed or completely occluded, causing a further aggravation of the lymphedema.

In response to exogenous stress due to the increase in endolymphatic pressure, smooth muscle cells in collecting lymphatic vessels undergo a transformation from “contractile” smooth muscle cells specialized in contractile functions to “synthetic” smooth muscle cells, which play an important part in the remodeling of the three-dimensional structure of the walls of the collecting lymphatic vessels. The smooth muscle cells that undergo transformation will proliferate and migrate again and will produce growth factors as well as enzymes that will degrade substrates in the extracellular matrix. Such smooth muscle functions are considered important in the healing mechanism against lymphatic vessel damage. However, if this process continues and becomes chronic, it tends to lead to the pathogenesis of diseases such as the hardening of the lymphatic vessels and to a decrease in their contractile force.

The “synthetic” smooth muscle cells migrate directly beneath the lymphatic vessel endothelium, where they proliferate and promote the formation of lesions characterized by the thickening of the lymphatic vessel wall. At this point, the changes are reversible, and fibrosis often develops when the disease continues and becomes chronic or when considerable inflammatory responses occur because of lymphangitis and cellulitis. These changes in collecting lymphatic vessels are termed lymphosclerosis (Figure 8).

The limitations of this study consist of the possibility of differences in the disease process between upper limb lymphedema and lower limb lymphedema. A further examination of this issue will be needed in the future. In addition, the association between the findings of this study and the effectiveness of treatment could also be used as a topic for further study. Moreover, the point at which a change is reversible or irreversible is still unclear.

This study has revealed that 1) the increase in endolymphatic pressure that develops after a lymphadenectomy is accompanied by histological changes in collecting lymphatic vessels and 2) these changes occur before the onset of lymphedema. It is important to prevent the aggravation of lymphedema by suppressing the irreversible degenerative changes in collecting lymphatic vessels by beginning therapeutic interventions at relatively early stages, such as at the dilation and contraction stages. The findings of this study could lead to the elucidation of the physiopathology of the onset of limb lymphedema after cancer treatment and to the development of a radical treatment.
