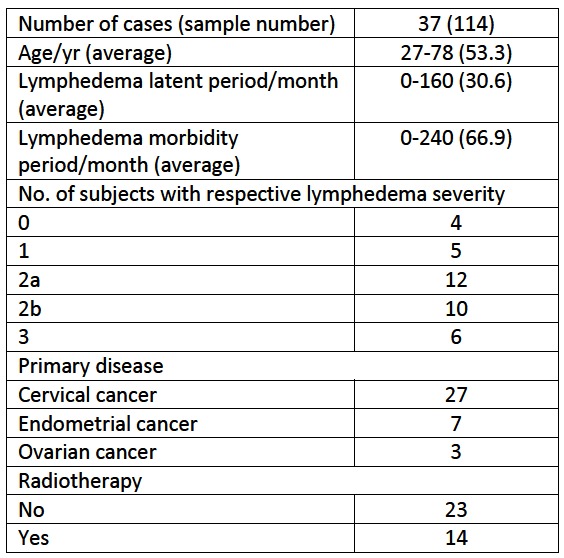# Correction: Pathological Steps of Cancer-Related Lymphedema: Histological Changes in the Collecting Lymphatic Vessels after Lymphadenectomy

**DOI:** 10.1371/annotation/6fff4d28-3f99-44eb-82d6-ccd885a1ba11

**Published:** 2013-05-22

**Authors:** Makoto Mihara, Hisako Hara, Yohei Hayashi, Mitsunaga Narushima, Takumi Yamamoto, Takeshi Todokoro, Takuya Iida, Naoya Sawamoto, Jun Araki, Kazuki Kikuchi, Noriyuki Murai, Taro Okitsu, Iori Kisu, Isao Koshima

There were errors in Table 1. The attache file contains a corrected version of the table: 

**Figure:**